# Are There Gender Differences in Coronary Artery Disease? The Malaysian National Cardiovascular Disease Database – Percutaneous Coronary Intervention (NCVD-PCI) Registry

**DOI:** 10.1371/journal.pone.0072382

**Published:** 2013-08-27

**Authors:** Chuey Yan Lee, Noran N. Hairi, Wan Azman Wan Ahmad, Omar Ismail, Houng Bang Liew, Robaayah Zambahari, Rosli Mohd Ali, Alan Yean Yip Fong, Kui Hian Sim, for the NCVD-PCI Investigators

**Affiliations:** 1 Department of Cardiology, Sultanah Aminah Hospital, Johor Bahru, Johor, Malaysia; 2 Department of Social and Preventive Medicine and Julius Centre University of Malaya, Faculty of Medicine, University Malaya, Petaling Jaya, Malaysia; 3 Division of Cardiology, University Malaya Medical Center, Petaling Jaya, Malaysia; 4 Department of Cardiology, Hospital Pulau Pinang, Penang, Malaysia; 5 Department of Cardiology, Queen Elizabeth Hospital, Sabah, Malaysia; 6 Department of Cardiology, National Heart Institute, Kuala Lumpur, Malaysia; 7 Department of Cardiology, Hospital Umum Sarawak, Sarawak, Malaysia; 8 Heart House, Level 1, Academy Medicine Building, Kuala Lumpur, Malaysia; University of Virginia Health System, United States of America

## Abstract

**Objectives:**

To assess whether gender differences exist in the clinical presentation, angiographic severity, management and outcomes in patients with coronary artery disease (CAD).

**Methods:**

The study comprised of 1,961 women and 8,593 men who underwent percutaneous coronary intervention (PCI) and were included in the Malaysian NCVD-PCI Registry from 2007–2009. Significant stenosis was defined as ≥70% stenosis in at least one of the epicardial vessels.

**Results:**

Women were significantly older and had significantly higher rates of diabetes mellitus, hypertension, chronic renal failure, new onset angina and prior history of heart failure whereas smokers and past history of myocardial infarction were higher in men. In the ST-elevation myocardial infarction (STEMI) cohort, more women were in Killip class III-IV, had longer door-to-balloon time (169.5 min. vs 127.3 min, p<0.052) and significantly longer transfer time (300.4 min vs 166.3 min, p<0.039). Overall, women had significantly more left main stem (LMS) disease (1.3% vs 0.6%, p<0.003) and smaller diameter vessels (<3.0 mm: 45.5% vs 34.8%, p<0.001). In-hospital mortality rates for all PCI, STEMI, Non-STEMI (NSTEMI) and unstable angina for women and men were 1.99% vs 0.98%, Odds ratio (OR): 2.06 (95% confidence interval (CI): 1.40 to 3.01), 6.19% vs 2.88%, OR: 2.23 (95% CI: 1.31 to 3.79), 2.90% vs 0.79%, OR: 3.75 (95% CI: 1.58 to 8.90) and 1.79% vs 0.29%, OR: 6.18 (95% CI: 0.56 to 68.83), respectively. Six-month adjusted OR for mortality for all PCI, STEMI and NSTEMI in women were 2.18 (95% CI: 0.97 to 4.90), 2.68 (95% CI: 0.37 to 19.61) and 2.66 (95% CI: 0.73 to 9.69), respectively.

**Conclusions:**

Women who underwent PCI were older with more co-morbidities. In-hospital and six-month mortality for all PCI, STEMI and NSTEMI were higher due largely to significantly more LMS disease, smaller diameter vessels, longer door-to-balloon and transfer time in women.

## Introduction

Cardiovascular disease is the leading cause of mortality in both men and women [1]. Each year more women than men die from cardiovascular disease, mostly from myocardial infarction and sudden death [1]. With the advancement of health care in general and cardiac care in particular, understanding possible gender-based differences in clinical characteristics, management and outcomes will help in improving current management of women with CAD.

Several studies had reported differences in clinical presentation and baseline characteristics of men and women [2]–[5]. Women were older and had higher rates of hypertension and diabetes but less likely to smoke tobacco then men [6]–[8]. Some studies [9]–[12], but not all [7], [8], had shown women with acute coronary syndrome (ACS) had worse in-hospital and long term prognoses then men. Data from the Global Registry of Acute Coronary Events (GRACE) [13], a large, multinational, observational study on patients with acute coronary syndrome who underwent coronary angiography showed that women were older than men and had higher rates of cardiovascular risk factor. Women were twice as likely to have normal/mild disease and less likely to have left-main and three vessel disease. Women with advanced disease had higher risk of in-hospital death. At six month, after adjustment for age and extent of disease, women were more likely to have adverse outcomes of death, myocardial infarction or rehospitalisation; however, sex differences in mortality were no longer statistically significant. Many studies had been carried out in developed countries; nevertheless information was sparse from the middle-income developing country like Malaysia.

The Malaysian NCVD-PCI Registry is a national observational study on a diverse, multi-ethnic population of real-world patients admitted for PCI.

Using data from the Malaysian NCVD-PCI Registry, we examined whether women admitted for PCI had different clinical presentation, severity of obstructive coronary artery disease, in-hospital and six-month outcome compared to men. Indeed, women have more co-morbidities and worse in-hospital and six-month outcomes.

## Methods

### Study Population

Full details of the Malaysian NCVD-PCI Registry has been described elsewhere “http://www.acrm.org.my/ncvd/pciReport_07-09.php” [14]. Briefly, the NCVD-PCI is an on-going observational prospective registry of patients who underwent PCI. It was started in 2007 and designed to evaluate the clinical presentation, angiographic severity, management and clinical outcomes of patients, 18 years and above with coronary artery disease who underwent PCI. This current report is based on the NCVD-PCI registry data collected from 1st January 2007 through 31st December 2009 from eleven participating centres. Data analysis was done annually and this is a 3-year pooled data. It comprised of 10,554 patients of which 1,961 (18.6%) were women and 8,593 (81.4%) were men with coronary artery disease. Women had 1,965 admissions and underwent 2,117 PCI compared to men who had 8,637 admissions and 9,381 PCI procedures.

### Study Variables

Data were collected regarding demographic characteristics, coronary risk factors (smoking, family history of premature cardiovascular disease, dyslipidemia, hypertension, diabetes mellitus) and other co-morbidities (Body Mass Index [BMI], history of myocardial infarct, new onset angina less than 2-weeks prior to admission, congestive heart failure more than 2-weeks prior to admission and chronic renal failure). Cardiac status at presentation, in-patient clinical care (time to treatment), types of adjunctive treatment used and angiographic severity were also captured. Normal/mild disease was defined as <50% stenosis and significant stenosis was defined as ≥70% stenosis in at least one of the epicardial vessels.

The outcomes of interest were in-hospital, 30-day and 6-month mortality post-PCI.

### Statistical Analysis

Statistical analysis was performed by describing demographic and clinical characteristics of the participants. T-tests and analysis of variance (ANOVA) were used to test differences in the distribution of continuous variables; the chi-square test was used to test for differences in the distribution of categorical variables. A multivariate logistic regression model was used to determine the risk association between variables identified and mortality for total population at discharge, 30 days and 6 months. Colinearity between variables was assessed before including in the final model. Subsequently, a multivariate logistic regression model was also developed to determine the association between gender and mortality at discharge, 30 days and 6 months. For each outcome variable, separate models were formulated for all PCI, STEMI patients, NSTEMI patients and those with unstable angina. Odds ratio and its 95% CI were calculated. The following sets of variables were included in the model: age, smoking, history of hypertension, diabetes mellitus, angina, heart failure and renal failure. For STEMI, Killip class at hospital admission (Killip class I as reference category) was included in the model. Variables with p<0.25 in the univariate analyses and variables of clinical significance were included in the models.

Statistical analyses were performed using STATA version 8 (Stata-Corp. College Station, TX, USA).

### Ethics Statement

The NCVD-PCI is registered in the National Medical Research Register of Malaysia (ID: NMRR-07-20-250) and received ethical approval from the Ministry of Health Medical Research and Ethics Committee.

## Results

### Patient Characteristics and Clinical Presentation

Women were significantly older than men but there were no significant difference in rate of premature CAD. Women had significantly higher rates of diabetes mellitus, hypertension, chronic renal failure, new onset angina and prior history of heart failure. Meanwhile, significantly more men were smokers and had past history of myocardial infarction. However, there were no significant difference in rates of BMI>26, dyslipidemia or documented coronary artery disease between gender. At presentation women had significantly more rapid heart rate and a higher blood pressure. In the STEMI cohort, more women were in Killip class III-IV (7.9% vs 5.3%, p = 0.047) (Table 1).

**Table 1:** Patients’ baseline characteristics and clinical presentation on admission by gender.

	Female(n = 1,965)	Male(n = 8,637)	All(n = 10,602)	*p*-value
Demographics				
Age, mean (SD), yrs	61.2 (9.7)	56.0 (10.0)	57.0 (10.2)	<0.001
Smoking status, n (%)				
Former	52 (2.7)	2,969 (34.4)	3,021 (28.5)	<0.001
Current	44 (2.2)	1,930 (22.4)	2,974 (18.6)	
BMI, n (%)				
Overweight and obese	1,369 (69.7)	5,963 (69.0)	7,332 (69.2)	0.245
Premature heart disease, n (%)	350 (17.8)	1,670 (19.3)	2,020 (19.1)	0.350
Clinical History, n (%)				
Diabetes	1,238 (63.0)	3,656 (42.3)	4,894 (46.2)	<0.001
Hypertension	1,685 (85.8)	6,116 (70.8)	7,801 (73.6)	<0.001
Chronic Renal Failure	183 (9.3)	517 (6.0)	700 (6.6)	<0.001
New onset angina (<2 weeks)	519 (26.4)	2,082 (24.1)	2,601 (24.5)	0.007
Congestive Heart Failure (>2 weeks)	90 (4.6)	333 (3.9)	423 (4.0)	0.011
Myocardial Infarction history	606 (30.8)	3,791 (43.9)	4,397 (41.5)	<0.001
Dyslipidemia	1,462 (74.4)	6,318 (73.2)	7,780 (73.4)	0.487
Documented Coronary Artery Disease	1,081 (55.0)	4,910 (56.9)	5,991 (56.5)	0.352
Clinical presentations				
Heart rate, mean (SD), beats/minute	73.8 (16.1)	71.1 (15.4)	71.6 (15.6)	<0.001
Systolic BP, mean (SD), mmHg	149.0 (28.8)	135.7 (24.8)	138.2 (26.1)	<0.001
Diastolic BP, mean (SD), mmHg	75.6 (13.2)	77.1 (13.0)	76.8 (13.0)	<0.001
Killip class (STEMI only), n (%)				
I & II	198 (58.4)	1,386 (60.5)	1584 (60.3)	0.047
III & IV	27 (7.9)	121 (5.3)	148 (5.6)	

*p*-values are calculated for gender, comparing all sub-categories except the unknown category for all variables.

All p-values are calculated using the Chi-square test unless stated.

We capture the time to treatment, when available, for STEMI patients presenting within 24 hours of symptom onset. Women had longer door-to-balloon time (mean 169.5 min. vs 127.3 min, p<0.052) and significantly longer transfer time (mean 300.4 min vs 166.3 min, p<0.039) although they had similar symptom-to-door time. Overall, women and men has similar rates of emergent (primary & rescue) STEMI PCI, but women receive less rescue PCI compared to men (36.8% vs 47.3%, p<0.035) although once a decision is made for rescue PCI higher percentage of women were done within 12 h (28.9% vs 20.9%, p<0.001) (Table 2). However, on further analysis of the STEMI PCI (primary, rescue and delayed) cohort alone (women n = 307, men n = 2,050), women in fact received less primary PCI (19.5% vs 23.6%, p = 0.0047) and the difference in the rescue PCI is no longer significant.

**Table 2:** In-patient clinical care by gender.

Type of treatment	Female(n = 1,965)	Male(n = 8,637)	All(n = 10,602)	*p*-value
PCI status, n (%)				
Elective	1,911 (90.3)	8,452 (90.1)	10,363 (90.1)	
NSTEMI/UA	106 (5.0)	420 (4.5)	526 (4.6)	
*STEMI	95 (4.5)	484 (5.2)	579 (5.0)	
(Rescue PCI	35 (36.8)†	229 (47.3)†	264 (45.6)†	0.035
Thrombolytics given prior to PCI procedure in STEMI, n (%)				
<12 hrs	42 (28.9)	95 (20.9)	112 (21.8)	<0.001

* patients who underwent PCI as emergent procedure (primary and rescue) during same index admission.

† As a percentage of total emergent PCI.

*p*-values are calculated for gender, comparing all sub-categories except the unknown catergory for all variables.

All p-values are calculated using the Chi-square test unless stated.

### Use of PCI Adjunct and Angiographic Disease Severity

These 10,554 patients underwent 11,498 PCI procedures, of these 2,117 (18.4%) were for women. Use of adjunctive pharmacotherapy viz. Glycoprotein IIb/IIIa inhibitor, heparin, aspirin and clopidogrel were similar for both gender. Overall, women and men had similar rate of single and multi-vessel disease, lesion type (A & B_1,_ B_2_ & C) and lesion length (≤20 mm, >20 mm) but women had significantly more left main stem disease (LMS) (1.3% vs 0.6%, p<0.003), smaller diameter vessel (<3.0 mm: 45.5% vs 34.8%, p<0.001) and received less drug eluting stent (39.8% vs 54.5%, p<0.001) (Table 3).

**Table 3:** The use of PCI adjunct and angiographic disease severity by gender.

	Female (n = 1,965)	Male (n = 8,637)	All (n = 10,602)	*p*-value
Adjunctive pharmacotherapy, n (%)				
IIb/IIa Blockade	118 (5.6)	571 (6.1)	689 (6.0)	0.286
Heparin	1,960 (92.6)	8,605 (91.7)	10,565 (91.9)	0.298
Aspirin	2,037 (96.2)	9,110 (97.1)	11,147 (97.0)	0.055
Clopidogrel	2,075 (98.0)	9203 (98.1)	11,278 (98.1)	0.778
Disease severity				
Coronary disease, n (%)				
Single vessel disease	983 (46.4)	4,318 (46.0)	5,301 (46.1)	0.370
Multiple vessel disease	1,119 (52.9)	4,991 (53.2)	6,110 (53.1)	0.401
Graft	17 (0.8)	113 (1.2)	130 (1.1)	0.036
Left Main Stem	27 (1.3)	55 (0.6)	82 (0.7)	0.003
Lesion type, n (%)				
A & B1	1.156 (39.3)	4,984 (38.5)	6,140 (38.7)	0.248
B2 & C	1,701 (57.9)	7,556 (58.4)	9,257 (58.4)	0.337
Lesion length, n (%), mm				
≤ 20	1,218 (41.5)	5,232 (40.5)	6,450 (40.7)	
>20	1,719 (58.5)	7,699 (59.5)	9418 (59.4)	0.200
Lesion diameter, n (%), mm				
< 3.0	1,337 (45.5)	4,503 (34.8)	5,840 (36.8)	
≤ 3.0	1,600 (54.5)	8,428 (65.2)	10,028 (63.2)	<0.001
Types of stent used, n (%)				
Drug Eluting Stent	339 (39.8)	2,289 (54.5)	2,628 (52.0)	
Bare Metal Stent	383 (45.0)	1,514 (36.0)	1,897 (37.5)	
Others	130 (15.2)	399 (9.5)	529 (10.5)	< 0.001

*p*-values are calculated for gender, comparing all sub-categories except the unknown catergory for all variables.

All p-values are calculated using the Chi-square test unless stated.

### In-hospital Mortality

Among the 10,554 patients who had PCI, 1,961 (18.6%) were women. Among these patients 2,357 presented with STEMI (13.0% women), 1,738 with NSTEMI (19.9% women), and 453 with unstable angina (24.7% women). In-hospital mortality risk for the total population was significantly increased in women (OR: 1.91 [95% CI: 1.06 to 3.46]), current smoker (OR: 2.62 [95% CI: 1.40 to 4.91]), diabetics (OR: 1.89 [95% CI: 1.17 to 3.04]), patients with new onset angina (OR: 2.27 [95% CI: 1.46 to 3.53]), presentation with Killip class III (OR: 6.80 [95% CI: 1.65 to 27.96]) and Killip class IV (OR: 61.40 [95% CI: 22.94 to 164.37]) Table 4). While in-hospital mortality rates for all PCI, STEMI, NSTEMI and unstable angina for women and men were 1.99% vs 0.98%, OR: 2.06 (95% CI: 1.40 to 3.01), 6.19% vs 2.88%, OR: 2.23 (95% CI: 1.31 to 3.79), 2.90% vs 0.79%, OR: 3.75 (95% CI: 1.58 to 8.9) and 1.79% vs 0.29%, OR: 6.18 (95% CI: 0.56–68.83), respectively. After adjusting for all significant confounding clinical characteristics viz. age, smoking, diabetes, hypertension, new onset angina, prior history of heart failure, chronic renal failure (and Killip class for STEMI cohort) OR were 1.71 (95% CI: 0.96 to 3.06), 1.06 (95% CI: 0.37 to 3.03), 2.70 (95% CI: 0.68 to 10.73) and 4.25 (95% CI:0.21 to 84.29), respectively Table 5). Using the Cox regression with forward variable selection, presentation with NSTEMI/unstable angina, STEMI and Killip class IV were significant prognostic factors for women whereas for men in addition to these, age ≥60 y, diabetes mellitus and Killip class II, and III were prognostically significant Table 6).

**Table 4:** Risk of mortality at discharge for total population

	All PCI	STEMI	NSTEMI	UA
Gender				
Female	1.91 (1.06, 3.46)	1.14 (0.40, 3.28)	2.90 (0.71, 11.81)	3.47 (0.21, 57.33)
Age (yr)				
	1.05 (1.03, 1.07)	1.05 (1.01, 1.09)	1.03 (0.98, 1.10)	1.30 (1.05, 1.63)
Smoking status				
Former	0.92 (0.47, 1.79)	0.34 (0.11, 1.08)	0.77 (0.13, 4.67)	-
Current	2.62 (1.40, 4.91)	1.32 (0.51, 3.42)	1.27 (0.21, 7.68)	-
HPT				
Yes	0.99 (0.56, 1.77)	0.97 (0.42, 2.24)	1.19 (0.24, 5.90)	-
DM				
Yes	1.89 (1.17, 3.04)	1.67 (0.78, 3.59)	2.01 (0.57, 7.12)	-
History of heart failure				
Yes	1.37 (0.88, 2.14)	0.81 (0.38, 1.70)	0.66 (0.20, 2.19)	0.96 (0.06, 16.41)
New onset of angina				
Yes	2.27 (1.46, 3.53)	1.58 (0.77, 3.25)	1.78 (0.58, 5.45)	0.54 (0.03, 8.92)
Renal failure				
Yes	1.44 (0.70, 2.96)	0.83 (0.17, 4.08)	1.82 (0.37, 9.11)	2.71 (0.15, 48.54)
Killip class				
II	-	2.55 (0.98, 6.66)	-	-
III	-	6.80 (1.65, 27.96)	-	-
IV	-	61.40 (22.94, 164.37)	-	-

Abbreviations: PCI  =  percutaneous coronary intervention; STEMI =  ST- elevation myocardial infarction; NSTEMI =  non-STEMI.

Odds ratio of total mortality and 95% CI obtained through logistic regression including the following covariates: Gender, age, smoking, hypertension, diabetes, prior history of heart failure, new onset of angina, renal failure and Killip class.

**Table 5:** In hospital mortality for women compared to men.

	Event Rates		Odds Ratio (95% Confidence Interval)	
	Women	Men	Crude	Adjusted
All PCI	39/1961 (1.99)	84/8593 (0.98)	2.06 (1.40, 3.01)	1.71 (0.96, 3.06)*
STEMI	19/307 (6.19)	59/2050 (2.88)	2.23 (1.31, 3.79)	1.06 (0.37, 3.03)†
NSTEMI	10/345 (2.90)	11/ 1393 (0.79)	3.75 (1.58, 8.90)	2.70 (0.68, 10.73)*
Unstable Angina	2/112 (1.79)	1 / 341 (0.29)	6.18 (0.56, 68.83)	4.25 (0.21, 84.29)*

Abbreviations: PCI  =  percutaneous coronary intervention; STEMI =  ST- elevation myocardial infarction; NSTEMI =  non-STEMI.

* Odds ratio of female vs male and 95% CI obtained through logistic regression including the following covariates: Age, smoking, diabetes, hypertension, new onset of angina, prior history of heart failure, renal failure.

† Odds ratio of female vs male and 95% CI obtained through logistic regression including the following covariates: Age, smoking, diabetes, hypertension, new onset of angina, prior history of heart failure, renal failure and Killip class.

**Table 6:** Prognostic factors for in-hospital mortality by gender

	Male				Female			
Variables	n	Hazard ratio	95% CI		n	Hazard ratio	95% CI	
Age group								
20 - <60	1,854	1.00						
≥60	962	2.92	(1.53, 5.58)					
PCI status								
Elective	2,337	1.00			464	1.00		
NSTEMI/UA	169	6.83	(2.18, 21.45)		38	8.30	(1.76, 39.06)	
STEMI	310	25.04	(10.83, 57.90)		64	15.09	(4.30, 52.93)	
Diabetes mellitus								
No	1,625	1.00						
Yes	1,191	2.02	(1.06, 3.85)					
Killip class								
I	1,803	1.00			360	1.00		
II	868	2.49	(1.08, 5.71)		165	1.38	(0.38, 4.96)	
III	79	6.73	(2.11, 21.52)		24	1.40	(0.15, 12.86)	
IV	66	21.20	(8.98, 50.03)		17	10.52	(2.79, 39.62)	

Abbreviations: PCI  =  percutaneous coronary intervention; STEMI =  ST- elevation myocardial infarction; NSTEMI =  non-STEMI.

### 30-day Mortality

At thirty days, 7,505 outcome data were available for analysis (3,049 were lost to follow-up), of which 1,330 (17.7%) were women. Among these patients 1,780 had STEMI (12.5% women), 1,366 had NSTEMI (19.0% women) and 332 had unstable angina (24.7% women). The 30-day mortality risk for the total population was significantly increased only in patients with chronic renal failure (OR: 3.65 [95% CI: 1.41 to 9.42]) while presentation with Killip class IV although elevated (OR: 12.72 [95% CI: 0.92 to 175.89]) did not reach statistical significance. All the other clinical factors were no longer significant Table 7). While the 30-day mortality rates for all PCI, STEMI and NSTEMI for women and men were 0.68% vs 0.39%, OR: 1.75 (95% CI: 0.81 to 3.77), 1.35 vs 0.39, OR: 3.54 (95% CI: 0.88 to 14.27) and 1.54 vs 0.45, OR: 3.44 (95% CI: 0.92 to 12.90), respectively. After adjustments for all significant confounding clinical characteristics OR were 1.08 (95% CI: 0.40 to 2.90), 1.7 (95% CI: 0.30 to 9.51) and 7.51 (95% CI: 0.94 to 60.21), respectively. The numbers for unstable angina were too small for analysis Table 8).

**Table 7:** Risk of mortality at 30 days for total population.

	All PCI	STEMI	NSTEMI	UA
Gender				
Female	1.10 (0.41, 2.94)	1.50 (0.10, 22.61)	7.41 (0.94, 58.51)	-
Age (yr)				
	1.05 (1.01, 1.09)	1.06 (0.97, 1.17)	1.01 (0.94, 1.09)	-
Smoking status				
Former	0.84 (0.32, 2.17)	1.10 (0.10, 11.75)	2.53 (0.27, 23.78)	-
Current	1.37 (0.47, 4.00)	0.66 (0.04, 11.03)	5.49 (0.66, 45.82)	-
HPT				
Yes	0.65 (0.27, 1.58)	0.46 (0.06, 3.80)	0.32 (0.07, 1.48)	-
DM				
Yes	1.18 (0.54, 2.60)	0.76 (0.10, 5.54)	0.87 (0.19, 4.07)	-
History of heart failure				
Yes	1.04 (0.50, 2.20)	0.71 (0.12, 4.74)	0.74 (0.17, 3.21)	-
New onset of angina				
Yes	0.81 (0.33, 2.00)	1.74 (0.27, 11.27)	0.57 (0.11, 2.90)	-
Renal failure				
Yes	3.65 (1.41, 9.42)	4.68 (0.42, 52.52)	-	-
Killip class				
II	-	1.39 (0.18, 10.59)	-	-
III	-	-	-	-
IV	-	12.72 (0.92, 175.89)	-	-

Abbreviations: PCI  =  percutaneous coronary intervention; STEMI =  ST- elevation myocardial infarction; NSTEMI =  non-STEMI.

Odds ratio of total mortality and 95% CI obtained through logistic regression including the following covariates: Gender, age, smoking, hypertension, diabetes, prior history of heart failure, new onset of angina, renal failure and Killip class.

**Table 8:** Risk of mortality at 30 days for women compared to men.

	Event Rates		Odds Ratio (95% Confidence Interval)	
	Women	Men	Crude	Adjusted
All PCI	9/1,330 (0.68)	24/6,175 (0.39)	1.75 (0.81, 3.77)	1.08 (0.40, 2.90)*
STEMI	3/222 (1.35)	6/1,558 (0.39)	3.54 (0.88, 14.27)	1.70 (0.30, 9.51)†
NSTEMI	4/260 (1.54)	5/1,106 (0.45)	3.44 (0.92, 12.90)	7.51 (0.94, 60.21)*
Unstable Angina	0/82 (0.00)	1 / 250 (0.40)	-	-

Abbreviations: PCI  =  percutaneous coronary intervention; STEMI =  ST- elevation myocardial infarction; NSTEMI =  non-STEMI.

* Odds ratio of female vs male and 95% CI obtained through logistic regression including the following covariates: Age, smoking, diabetes, hypertension, new onset of angina, prior history of heart failure, renal failure.

† Odds ratio of female vs male and 95% CI obtained through logistic regression including the following covariates: Age, smoking, diabetes, hypertension, new onset of angina, prior history of heart failure, renal failure and Killip class.

### 6-month Mortality

At 6 months 5,495 outcome data were available for analysis (2,010 were loss to follow-up), of which 987 (18%) were women. Among these patients 1,338 had STEMI (12.6% women), 1,044 had NSTEMI (19.3% women) and 247 unstable angina (23.9% women). The 6-month mortality risk for the total population was elevated in female (OR: 2.2 [95% CI: 0.98 to 4.91]), diabetics (OR 2.22 [95% CI: 0.97 to 5.05]) and patients with chronic renal failure (OR: 2.44 [95% CI: 0.96 to 6.20]) but these did not reach statistical significance Table 9). While the 6-month mortality rates for all PCI, STEMI and NSTEMI for women and men were 1.93 vs 0.42, OR: 4.64 (95% CI: 2.45 to 8.79), 1.78 vs 0.43, OR: 4.21 (95% CI: 1.00 to 17.77) and 2.48 vs 0.59, OR: 4.25 (95% CI: 1.22 to 14.82), respectively. After adjustments for all significant confounding clinical characteristics OR were 2.18 (95% CI: 0.97 to 4.90), 2.68 (95% CI: 0.37 to 19.61) and 2.66 (95% CI: 0.73 to 9.69), respectively Table 10).

**Table 9:** Risk of mortality at 6 months for total population

	All PCI	STEMI	NSTEMI	UA
Gender				
Female	2.20 (0.98, 4.91)	1.28 (0.11, 15.30)	1.69 (0.37, 7.71)	-
Age (yr)				
	1.04 (1.00, 1.08)	1.00 (0.91, 1.09)	1.12 (1.03, 1.22)	-
Smoking status				
Former	0.37 (0.12, 1.18)	0.44 (0.03, 6.45)	0.35 (0.04, 3.29)	-
Current	0.41 (0.09, 1.88)	0.26 (0.02, 3.75)	-	-
HPT				
Yes	0.79 (0.28, 2.19)	0.15 (0.02, 1.14)	1.40 (0.16, 12.61)	-
DM				
Yes	2.22 (0.97, 5.05)	8.59 (0.80, 92.27)	1.08 (0.24, 4.88)	-
History of heart failure				
Yes	0.82 (0.39, 1.74)	0.14 (0.01, 1.43)	0.78 (0.18, 3.46)	-
New onset of angina				
Yes	1.55 (0.73, 3.27)	2.63 (0.35, 19.86)	0.97 (0.22, 4.30)	-
Renal failure				
Yes	2.44 (0.96, 6.20)	-	0.91 (0.08, 9.74)	-
Killip class				
II	-	0.54 (0.05, 5.54)	-	-
III	-	-	-	-
IV	-	-	-	-

Abbreviations: PCI  =  percutaneous coronary intervention; STEMI =  ST- elevation myocardial infarction; NSTEMI =  non-STEMI.

Odds ratio of total mortality and 95% CI obtained through logistic regression including the following covariates: Gender, age, smoking, hypertension, diabetes, prior history of heart failure, new onset of angina, renal failure and Killip class.

**Table 10:** Risk of mortality at 6 months for women compared to men.

	Event Rates		Odds Ratio (95% Confidence Interval)	
	Women	Men	Crude	Adjusted
All PCI	19/987 (1.93)	19/4,508 (0.42)	4.64 (2.54, 8.79)	2.18 (0.97, 4.90)*
STEMI	3/169 (1.78)	5/ 1,169 (0.43)	4.21 (1.00, 17.77)	2.68 (0.37, 19.61)†
NSTEMI	5/202 (2.48)	5/842 (0.59)	4.25 (1.22, 14.82)	2.66 (0.73, 9.69)*
Unstable Angina	1/59 (1.69)	0/188 (0.00)	-	-

Abbreviations: PCI  =  percutaneous coronary intervention; STEMI =  ST- elevation myocardial infarction; NSTEMI =  non-STEMI.

* Odds ratio of female vs male and 95% CI obtained through logistic regression including the following covariates: Age, smoking, diabetes, hypertension, new onset of angina, prior history of heart failure, renal failure.

† Odds ratio of female vs male and 95% CI obtained through logistic regression including the following covariates: Age, smoking, diabetes, hypertension, new onset of angina, prior history of heart failure, renal failure and Killip class.

## Discussion

In this cohort of men and women who underwent percutaneous coronary intervention, gender differences were observed in baseline characteristics, clinical presentation, extent of angiographic disease, management and in-hospital and six-month outcomes.

### Baseline Characteristics and Clinical Presentation

The study comprised of 10,554 patients, 1961 (18.6%) were women and 8,593 (81.4%) were men with a female to male ratio of 0.23. While in the Malaysian National Cardiovascular Disease Database-Acute Coronary Syndrome (NCVD-ACS) Registry, the female to male ratio for patients admitted for acute coronary syndrome (ACS) for the period 2007–2009 was 0.32 (2,415∶7,658) [15], [16]. This is in contrast to the general Malaysian population which has a female to male ratio of 0.95 (13,771,497∶14,562,638) according to the year 2010 population census [17]. This lower incidence of PCI in women could partly be due to refusal to undergo invasive procedure and general lack of awareness among women about coronary artery disease, thus failure to sought treatment. Women present with coronary artery disease 5 years later than men. This is due to the protective effect of oestrogen. Upon reaching menopause the incidence of coronary artery disease catches up with that of men. This 5 years delay could partly explain why women has higher incidence of co-morbidities.

Some of the results of this study were similar to the GRACE registry [13] whereby among those who had a cardiac catheterization, women had higher rates of diabetes, hypertension, prior angina and heart failure, but were less likely to smoke tobacco or have a history of myocardial infarction. In addition this study showed women also had higher rate of chronic renal failure. In the STEMI cohort, women had longer door-to-balloon time and transfer time. This could partly be due to cultural influence whereby Asian men are usually head of the household and also the decision maker in the family. Usually women patient would wait for their spouse to agree before any consent for PCI was given. Inertia of physician to refer from non-PCI centers may also be responsible although once the decision to proceed for PCI were made; there were no delay by the interventionalists.

### Use of PCI Adjunct and Angiographic Disease Severity

With regards to use of PCI adjunct, findings of this study differ from the GRACE registry [13]. Women received similar rates of adjunctive pharmacotherapy whereas in the GRACE registry women with ACS received less glycoprotein IIb/IIIa inhibitors. Also, in this study women had comparable rates of single and multi-vessel disease but higher rates of left main stem disease and smaller vessel diameter. However, in the Grace registry women were twice as likely to have normal/mild disease and were less likely to have three-vessel or left main stem disease although they were not directly comparable as GRACE registry included only ACS patients undergoing catheterization. Another interesting finding was that women received less drug-eluting stent. In Malaysia patients had to pay for the drug-eluting stents whilst use of bare-metal stents were fully subsided by the government in the public hospitals. This difference could again partly be due to cultural influence whereby men being the head of the household and usually the bread winner for the family occupy a more important position in the family whereas women are usually housewives and thus being deemed as less important. Thus, the family may be less willing to part with their money. Furthermore, the risk of in-stent restenosis increased with the use of bare metal stent (10%–60%) compared to drug-eluting stent (<10%) [18]. This would affect the 30-day and 6-month outcome as in-stent restenosis could result in recurrent angina, NSTEMI, STEMI or death depending on the site and severity of the restenosis. This is an important issue independent of the gender-based differences in physiology which the Malaysian health-care providers need to tackle. There is a need to impress upon the policy-makers in particular and the population in general regarding the cost-effectiveness of drug-eluting stent.

### In-hospital Mortality

Women had significantly higher unadjusted in-hospital mortality for all PCI, STEMI and NSTEMI. This was similar to the GRACE registry [13] where in-hospital death rate were 4.5 (339/7,613) for women and 2.6% (496/19,078) for men. The higher mortality could largely be explained by more LMS disease, smaller vessels, longer door-to-balloon and transfer time in women. After multivariable adjustment for all significant clinical characteristics, there was still a trend towards higher mortality for all PCI and NSTEMI in women although these differences were no longer statistically significant.

### 30-day Mortality

At 30 days there was still a trend towards higher unadjusted mortality rates for all PCI, STEMI and NSTEMI in women although not significant. After multivariable adjustment these differences were much attenuated except for NSTEMI where the rate was increased, but all these differences were not significant. Jeffrey S. Berger et al [19] in the paper “Sex Differences in Mortality Following Acute Coronary Syndromes” using data pooled from 11 independent, international, randomised ACS clinical trials between 1993 and 2006 involving 136,247 patients (38,048 [28%] women) whose data bases are maintained at the Duke Clinical research institute, Durham, North Carolina found that women had a significantly higher unadjusted 30-day mortality compared to men (OR: 1.91 [95% CI: 1.83 to 2.00]). After multivariable adjustment for clinical characteristics, no significant differences was observed (OR: 1.06 [95% CI: 0.99 to 1.15]). Among patients with STEMI, 30-day mortality was significantly higher among women compared to men, however it was markedly attenuated after adjustment (unadjusted OR: 2.29 [95% CI: 2.18 to 2.40], adjusted OR: 1.15 [95% CI: 1.06–1.24]). In contrast the unadjusted risk in NSTEMI was significantly higher in women compared to men but after adjustment 30-day mortality was lower in women (unadjusted OR: 1.50 [95% CI: 1.28 to 1.75], adjusted OR: 0.77[95% CI: 0.63 to 0.95]). In unstable angina men and women had similar unadjusted risk, however after adjustment; women had a significantly lower mortality than men. Our sample of patients was small. If we continue the data collection over a longer duration involving a larger number of patients we may see a similar result. However, we must bear in mind the data by Jeffrey S. Berger et al [19] were based on clinical trial patients whereas our cohort was real-world all-comers for PCI. Thus, our data may be more representative of existing gender differences.

### 6-month Mortality

At 6 months women had significantly higher unadjusted mortality rates for all PCI, STEMI and NSTEMI. After multivariable adjustment for all significant clinical characteristics, there was still a trend towards higher mortality rates but these differences were no longer significant. In the GRACE registry [13], women with advanced CAD were more likely to have adverse outcomes (death, myocardial infarction, stroke and rehospitalisation) at six months compared to men (25% vs 20%, p<0.001). These differences remained after adjustment for age and number of diseased vessels (OR: 1.25 [95% CI: 1.14 to 1.34]); however, differences in death rates were no longer significant between women and men after these adjustments (OR: 0.97 [95% CI: 0.81 to 1.15]). Results of this study were similar to the GRACE registry [13] although in the Grace registry adjustment was only made for age and extend of disease whereas this study made adjustment for age, smoking, hypertension, diabetes mellitus, new onset angina, recent history of heart failure, renal failure and Killip class (in STEMI cohort).

### Strength and Limitations

The Malaysian NCVD-PCI registry has the advantage of being a ‘real-world’ study that provided data on a multi-ethnic, multi-cultural population. It also included patients who were often under-represented in randomised trials. The outcome data made adjustment for most of the important and well-known coronary risk factors and co-morbidities known to us today, giving a better estimate of mortality. However, as a non-randomised observational study it is subjected to certain limitations and potential bias. As a result of movement or transfer of patients from their home either due to work or personal reasons, some of the patients were lost to follow-up. However, as far as possible we tried to correct this by cross checking with the National Registration Department to confirm if patients were still alive at each follow-up period. Since the data in this study were limited to patients who underwent percutaneous coronary intervention, it may not be possible to generalise these finding to all patients with coronary artery disease. Angiographic interpretation was based on semi-quantitative clinical report, not on the results of a blinded core laboratory and/or quantitative coronary angiography interpretation. Also, study population was small leading to a wide range in confidence intervals. If we are able to continue this registry for the years to come and hopefully with more funding and support in terms of human resource we may be able to get a better picture. Collected data were reviewed annually and new technology or data deemed important were added and obsolete or unimportant data were excluded from the database periodically.

## Conclusion

Gender differences in mortality have been a major topic of study during the past decades. Despite these studies the relationship is poorly understood. Some studies showed increased rates of mortality, some no difference, while others showed lower rates of mortality for women compared to men. Our study involving a multi-ethnic, multi-cultural group of patients with CAD found that women were older and more likely to have diabetes mellitus, hypertension, chronic renal failure, new onset angina and prior history of heart failure whereas smokers and past history of myocardial infarction were higher in men. More women with STEMI present with Killip class III to IV heart failure. In-hospital and six-month mortality were significantly higher in women compared to men for all PCI, STEMI and NSTEMI although these differences were no longer significant once adjustment for all these con-founding factors were made. The higher mortality could largely be explained by more LMS disease, smaller vessels, longer door-to-balloon and transfer time in women. These findings suggested that we need to be more vigilant and aggressive in the management of all co-morbidities in women if our attempt to improve outcome is to succeed. Further research regarding possible gender differences in disease severity will assist us in prevention and management of CAD.
